# Supplementary material for: Reverse Vaccinology Approach to Identify Novel and Immunogenic Targets against Streptococcus gordonii
Source: Biology (Basel). 2024 Jul 9;13(7):510. doi: 10.3390/biology13070510 (PMC11274250; doi:10.3390/biology13070510)
Supplement: Supplementary file 1 [file biology-13-00510-s001.zip › biology-3032040-supplementary.pdf]

Table S1. Antigenicity, Allergenicity, Toxicity, and Sub-Cellular Localization of Selected MHC-I Restricted Epitopes

Protein	Sequence/ Epitope	Antigenicity	Allergenicity	Toxicity	Sub-cellular localization
YSIRK_signal domain-containing protein	FYYPPFPDM	1.9204	Non-allergen	Non-toxic	Outside
	REPFYYPPF	1.3015	Non-allergen	Non-toxic	Outside
	VQVDSVTEE	1.4495	Non-allergen	Non-toxic	Outside
Peptidoglycan D,D-transpeptidase FtsI	WAVVLLCFV	1.2845	Non-allergen	Non-toxic	Outside
	VLLCFVVLI	1.5192	Non-allergen	Non-toxic	Outside
	LWAVVLLCF	1.5198	Non-allergen	Non-toxic	Outside
	AVVLLCFVV	1.4047	Non-allergen	Non-toxic	Outside

Table S2. Physio-Chemical Properties of MHC-I Restricted Epitopes

Protein	Sequence/ Epitope	Hydrophobicity	Hydrophilicity	Charge	pI	MW
YSIRK_signal domain-containing protein	FYYPPFPDM	0.07	-0.88	-1	3.8	1176.35
	REPFYYPPF	-0.15	-0.4	0	6.35	1215.37
	VQVDSVTEE	-0.16	0.51	-3	3.58	1005.05
Peptidoglycan D,D-transpeptidase FtsI	WAVVLLCFV	0.44	-1.72	0	5.85	1049.34
	VLLCFVVLI	0.51	-1.69	0	5.85	1018.37

	LWAVVLL CF	0.44	-1.76	0	5.85	1063.37
	AVVLLCFV V	0.46	-1.51	0	5.85	962.26

Table S3. Non-Digesting Enzymes of MHC-I Restricted Epitopes

**Protein**	**Sequence/ Epitope**	**Non-digesting Enzymes**
**YSIRK_signal domain-containing protein**	FYYPPFPDM	Arg-C proteinase, BNPS-Skatole, Caspase1-10, Clostripain, Enterokinase, Factor Xa, Glutamyl endopeptidase, GranzymeB, Hydroxylamine, Iodosobenzoic acid, LysC, LsyN, NTCB (2-nitro-5-thiocyanobenzoic acid), Proline-endopeptidase, Staphylococcal peptidase I, Thermolysin, Thrombin, Tobacco etch virus protease, Trypsin
	REPFYYPPF	Asp-N endopeptidase, BNPS-Skatole, CNBr, Caspase1-10, Enterokinase, Factor Xa, Formic acid, GranzymeB, Hydroxylamine, Iodosobenzoic acid, LysC, LsyN, NTCB (2-nitro-5-thiocyanobenzoic acid), Pepsin (pH1.3), Pepsin (pH>2), Proline-endopeptidase, Thrombin, Tobacco etch virus protease
	VQVDSVTEE	Arg-C proteinase, BNPS-Skatole, CNBr, Caspase1-10, Chymotrypsin-high specificity (C-term to [FYW], not before P), Chymotrypsin-low specificity (C-term to [FYWL], not before P), Clostripain, Enterokinase, Factor Xa, GranzymeB,

		Hydroxylamine, Iodosobenzoic acid, LysC, LsyN, NTCB (2-nitro-5-thiocyanobenzoic acid), Pepsin (pH1.3), Pepsin (pH>2), Proline-endopeptidase, Thrombin, Tobacco etch virus protease, Trypsin
**Peptidoglycan D,D-transpeptidase FtsI**	WAVVLLCFV	Arg-C proteinase, Asp-N endopeptidase, Asp-N endopeptidase + N-terminal Glu, CNBr, Caspase1-10, Clostripain, Enterokinase, Factor Xa, Formic acid, Glutamyl endopeptidase, GranzymeB, Hydroxylamine, LysC, LsyN, Proline-endopeptidase, Staphlococcal peptidase I, Thrombin, Tobacco etch virus protease, Trypsin
	VLLCFVVLI	Arg-C proteinase, Asp-N endopeptidase, Asp-N endopeptidase + N-terminal Glu, BNPS-Skatole, CNBr, Caspase1-10, Clostripain, Enterokinase, Factor Xa, Formic acid, Glutamyl endopeptidase, GranzymeB, Hydroxylamine, Iodosobenzoic acid, LysC, LsyN, Proline-endopeptidase, Staphlococcal peptidase I, Thrombin, Tobacco etch virus protease, Trypsin
	LWAVVLLCF	Arg-C proteinase, Asp-N endopeptidase, Asp-N endopeptidase + N-terminal Glu, CNBr, Caspase1-10, Clostripain, Enterokinase, Factor Xa, Formic acid, Glutamyl endopeptidase, GranzymeB, Hydroxylamine, Iodosobenzoic acid, LysC, LsyN, Proline-endopeptidase, Staphlococcal peptidase I, Thrombin, Tobacco etch virus protease, Trypsin

	AVVLLCFVV	Arg-C proteinase, Asp-N endopeptidase, Asp-N endopeptidase + N-terminal Glu, BNPS-Skatole, CNBr, Caspase1-10, Clostripain, Enterokinase, Factor Xa, Formic acid, Glutamyl endopeptidase, GranzymeB, Hydroxylamine, Iodosobenzoic acid, LysC, LsyN, Proline-endopeptidase, Staphlococcal peptidase I, Thrombin, Tobacco etch virus protease, Trypsin

Table S4. Physio-chemical properties of MHC-II Restricted Epitopes

**Protein**	**Sequence/ Epitope**	**Hydrophobicity**	**Hydrophilicity**	**Charge**	**pI**	**MW**
**YSIRK_signal domain-containing protein**	APFVFKPESTPAPKL	-0.05	-0.03	1	8.94	1628.93
	PFVFKPESTPAPKLD	-0.11	0.21	0	6.42	1672.94
	FVFKPESTPAPKLDM	-0.09	0.12	0	6.42	1707.02
**Peptidoglycan D,D-transpeptidase FtsI**	PGENITLSIDSRLQY	-0.17	0.01	-1	4.38	1705.89
	GTMAYGYGLNATILQ	0.09	-0.85	0	5.87	1572.8
	VLLCFVVLIARAFYV	0.3	-1.3	1	8.57	1726.2
	AQIIGLTNSEGQGIE	-0.02	-0.08	-2	3.8	1529.67

Table S5. Non-digesting enzymes of MHC-II Restricted Epitopes

Protein	Sequence/ Epitope	Non-digesting Enzymes
**YSIRK_signal domain-containing protein**	APFVFKPESTPAPKL	Arg-C proteinase, Asp-N endopeptidase, BNPS-Skatole, CNBr, Caspase1-10, Clostripain, Enterokinase, Factor Xa, Formic acid, GranzymeB, Hydroxylamine, Iodosobenzoic acid, NTCB (2-nitro-5-thiocyanobenzoic acid), Thrombin, Tobacco etch virus protease
	PFVFKPESTPAPKLD	Arg-C proteinase, BNPS-Skatole, CNBr, Caspase1-10, Clostripain, Enterokinase, Factor Xa, GranzymeB, Hydroxylamine, Iodosobenzoic acid, NTCB (2-nitro-5-thiocyanobenzoic acid), Thrombin, Tobacco etch virus protease
	FVFKPESTPAPKLD M	Arg-C proteinase, BNPS-Skatole, Caspase1-10, Clostripain, Enterokinase, Factor Xa, GranzymeB, Hydroxylamine, Iodosobenzoic acid, NTCB (2-nitro-5-thiocyanobenzoic acid), Thrombin. Tobacco etch virus protease
**Peptidoglycan D,D-transpeptidase FtsI**	PGENITLSIDSRLQY	BNPS-Skatole, CNBr, Caspase1-10, Enterokinase, Factor Xa, GranzymeB, Hydroxylamine, Iodosobenzoic acid, LysC, LsyN, NTCB (2-nitro-5-thiocyanobenzoic acid), Proline-endopeptidase, Thrombin. Tobacco etch virus protease
	GTMAYGYGLNATIL Q	Arg-C proteinase, Asp-N endopeptidase, Asp-N endopeptidase + N-terminal Glu, BNPS-Skatole, Caspase1-10, Clostripain, Enterokinase, Factor Xa, Formic acid, Glutamyl endopeptidase, GranzymeB, Hydroxylamine, Iodosobenzoic acid, LysC, LsyN, NTCB (2-nitro-5-thiocyanobenzoic acid), Proline-endopeptidase, Staphylococcal peptidase I, Thrombin. Tobacco etch virus protease, Trypsin

	VLLCFVVLIARAFYV	Asp-N endopeptidase, Asp-N endopeptidase + N-terminal Glu, BNPS-Skatole, CNBr, Caspase1-10, Clostripain, Enterokinase, Factor Xa, Formic acid, Glutamyl endopeptidase, GranzymeB, Hydroxylamine, Iodosobenzoic acid, LysC, LsyN, Proline-endopeptidase, Staphylococcal peptidase I, Thrombin, Tobacco etch virus protease
	AQIIGLTNSEGQGIE	Arg-C proteinase, Asp-N endopeptidase, BNPS-Skatole, CNBr, Caspase1-10, Chymotrypsin-high specificity (C-term to [FYW], not before P), Clostripain, Enterokinase, Factor Xa, Formic acid, Glutamyl endopeptidase, GranzymeB, Hydroxylamine, Iodosobenzoic acid, LysC, LsyN, NTCB (2-nitro-5-thiocyanobenzoic acid), Proline-endopeptidase, Staphylococcal peptidase I, Thrombin, Tobacco etch virus protease, Trypsin

Table S6. Conservation analysis of epitopes of YSIRK_signal domain protein computed via IEDB epitope conservancy analysis tool

Epitope Sequence	Epitope Length	Percentage of Sequence matches at identity <= 100%	Minimum Identity	Maximum Identity
LWTPNGLTKGNENNAP	16	0.00% (0/1)	31.25%	31.25%
FYYPPFPDM	9	0.00% (0/1)	33.33%	33.33%
REPFYYPPF	9	0.00% (0/1)	44.44%	44.44%

VQVDSVTEE	9	0.00% (0/1)	55.56%	55.56%
APFVFKPESTPAPKL	15	0.00% (0/1)	40.00%	40.00%
PFVFKPESTPAPKLD	15	0.00% (0/1)	33.33%	33.33%
FVFKPESTPAPKLDM	15	0.00% (0/1)	33.33%	33.33%

Table S7. Conservation analysis of epitopes of Peptidoglycan D, D-transpeptidase FtsI computed via IEDB epitope conservancy analysis tool

Epitope Sequence	Epitope Length	Percentage of Sequence matches at identity <= 100%	Minimum Identity	Maximum Identity
FDMWRFYLLWAVVLLC	16	0.00% (0/1)	31.25%	31.25%
EPGENITLSIDSRLQY	16	0.00% (0/1)	37.50%	37.50%
WAVVLLCFV	9	0.00% (0/1)	44.44%	44.44%
VLLCFVVLI	9	0.00% (0/1)	66.67%	66.67%
LWAVVLLCF	9	0.00% (0/1)	44.44%	44.44%
AVVLLCFVV	9	0.00% (0/1)	44.44%	44.44%
PGENITLSIDSRLQY	15	0.00% (0/1)	40.00%	40.00%
GTMAYGYGLNATILQ	15	0.00% (0/1)	33.33%	33.33%
VLLCFVVLIARAFYV	15	0.00% (0/1)	46.67%	46.67%
AQIIGLTNSEGQGIE	15	0.00% (0/1)	33.33%	33.33%

Table S8. Physio-chemical Properties and Solubility of Multivalent Vaccine

Sr. #	Property	Vaccine with adjuvant and EAAK linker	Vaccine without adjuvant and EAAK linker
**1**	**Molecular weight**	44750.94kDa	30412.33kDa
**2**	**Number of amino acids**	410	282
**3**	**pI value**	7.12	6.07

**4**	**Instability index**	36.80	38.88
**5**	**Aliphatic index**	88.78	88.55
**6**	**Gravy**	0.135	0.246
**7**	**Half life estimation**	In vitro: 30 hours (mammalian reticulocytes) In vivo: > 20 hours (yeast) and > 10 hours (E.coli)	In vitro: 5.5 hours (mammalian reticulocytes) In vivo: 3 min (yeast) and 2 min (E. coli)
**8**	**Protein solubility**	0.837	0.289
